# Metastatic Bifocal Germinoma With Dramatic Early Steroid Response, Utility of Circulating miR‐371a‐3p and Vinblastine Monotherapy Prior to Definitive Craniospinal Irradiation

**DOI:** 10.1111/nan.70030

**Published:** 2025-07-05

**Authors:** Cinzia G. Scarpini, Dawn Ward, Poe Phyu, Ibrahim Jalloh, Nicky Thorp, Benjamin G. Fisher, A. Emile J. Hendriks, Lyndsay Salisbury, James C. Nicholson, Thankamma Ajithkumar, Gail Horan, G. A. Amos Burke, Nicholas Coleman, Kieren Allinson, Matthew J. Murray

**Affiliations:** ^1^ Department of Pathology University of Cambridge Cambridge UK; ^2^ Department of Radiology Cambridge University Hospitals NHS Foundation Trust Cambridge UK; ^3^ Department of Neurosurgery Cambridge University Hospitals NHS Foundation Trust Cambridge UK; ^4^ Department of Radiation Oncology The Christie Proton Beam Therapy Centre Manchester UK; ^5^ Department of Paediatric Endocrinology Cambridge University Hospitals NHS Foundation Trust Cambridge UK; ^6^ Department of Paediatric Haematology and Oncology Cambridge University Hospitals NHS Foundation Trust Cambridge UK; ^7^ Department of Radiation Oncology Cambridge University Hospitals NHS Foundation Trust Cambridge UK; ^8^ Cancer Research UK Clinical Trials Unit (CRCTU) School of Medical Sciences, University of Birmingham, Edgbaston Birmingham UK; ^9^ Department of Neuropathology Cambridge University Hospitals NHS Foundation Trust Cambridge UK

**Keywords:** cytokine, dexamethasone, germ cell tumour, germinoma, lymphocyte, miR‐371a‐3p

Central nervous system (CNS) germ cell tumours (GCTs) are rare and heterogeneous due to different anatomical sites, subtypes and variable serum and cerebrospinal fluid (CSF) alpha‐fetoprotein (AFP) and human chorionic gonadotrophin (HCG) marker expression [1]. As a result, diagnosis and management are challenging [1]. CNS GCTs are typically segregated into pure germinoma (two‐thirds of cases) and the more aggressive non‐germinomatous GCTs (NGGCTs), the latter needing more intensive therapy for cure [1]. In Europe, CNS pure germinomas are diagnosed by both serum and CSF AFP and HCG levels being below specific thresholds [25 ng/mL (20.8 kU/L) and 50 IU/L, respectively] and histological confirmation [1]. The exception is for ‘bifocal’ germinoma (occurring in both the pineal and suprasellar regions), which can be diagnosed without recourse to biopsy where clinical presentation is typical [patient age > 8–10 years of age and central diabetes insipidus (DI) present], along with serum and CSF AFP and HCG below threshold values and with expected radiological findings [2, 3]. In such cases, a diagnosis of germinoma can be assumed, and patients treated accordingly [2, 3]. If there is any diagnostic uncertainty, a low threshold for considering biopsy must be maintained, as very rarely, NGGCT may similarly be present [3]. Importantly, in a recent large bifocal cohort of 89 cases, no tumours other than GCTs were observed, and although three NGGCT cases were identified, none had a full set of serum/CSF AFP/HCG markers [4]. Thus, in patients with complete tumour marker work‐up, only pure germinoma was identified [4].

CNS germinomas are typically characterised by a profound lymphocytic infiltration, making diagnosis challenging, as malignant cells may be sparse [3]. Accordingly, dramatic radiological responses to steroids (dexamethasone/methylprednisolone) have been noted in such cases [5, 6, 7], presumed secondary to immunosuppressive effects. Caution is therefore required in over‐interpreting early radiological responses in such cases. Recently, novel intracranial malignant GCT biomarkers have been reported, such as CSF placental alkaline phosphatase (PLAP) for germinoma [8], and circulating microRNAs, particularly miR‐371a‐3p, for malignant GCTs [9, 10], including intracranial disease [11, 12, 13]. Here, we report an informative metastatic bifocal germinoma case highlighting the importance of being aware of this steroid‐induced phenomenon and the utility of both circulating miR‐371a‐3p quantification [11, 12, 13] and vinblastine monotherapy [11, 14] prior to definitive radiotherapy.

A 12‐year‐old boy presented with a 4‐month history of headaches and a 3‐month history of polyuria and polydipsia, visual impairment, somnolence, and memory issues. Examination revealed a confused and drowsy patient, but no specific neurological deficit. Auxology showed a weight of 71.1 kg (99.6th centile) and a height of 166.0 cm (98.9th centile). MRI demonstrated hydrocephalus and a solid, enhancing 19 × 14 × 15 mm diameter nodule in the pineal region (volume 2.09 cm^3^) (Figure 1A), with nodular soft tissue in the suprasellar (Figure 1B) and septum pellucidum (Figure 1C) regions, but no other intracranial or spinal disease on imaging. An emergency extraventricular drain (EVD) was placed to manage the hydrocephalus and raised intracranial pressure (RICP) alongside administration of dexamethasone (8 mg/day = 4.4 mg/m^2^/day). CSF cytology (taken upon EVD placement) revealed scattered large malignant cells with irregular hyperchromatic nuclei. Serum and CSF AFP (both < 4 kU/L, range 0–7) and HCG (serum < 2 IU/L, range 0–2; CSF < 10 IU/L, range < 10) were within institutional reference ranges (i.e., negative). Further investigations confirmed central DI and hypothyroidism, for which DDAVP and replacement thyroxine were commenced. A diagnosis of metastatic bifocal germinoma was made without need for biopsy [3], and plans were made for definitive treatment with 24 Gy craniospinal irradiation (CSI) with a 16 Gy boost to macroscopic disease, as per current European recommendations [3]. The expected time from presentation to start of proton therapy was estimated to be approximately 8 weeks. Accordingly, and given the exquisite sensitivity of germinoma to chemotherapy, weekly intravenous vinblastine monotherapy was commenced as a ‘bridge’ to radiotherapy to prevent the development of further co‐morbidities in the interim [14] and with the expectation that the EVD would be removed without need for further neurosurgical intervention.

**FIGURE 1:**
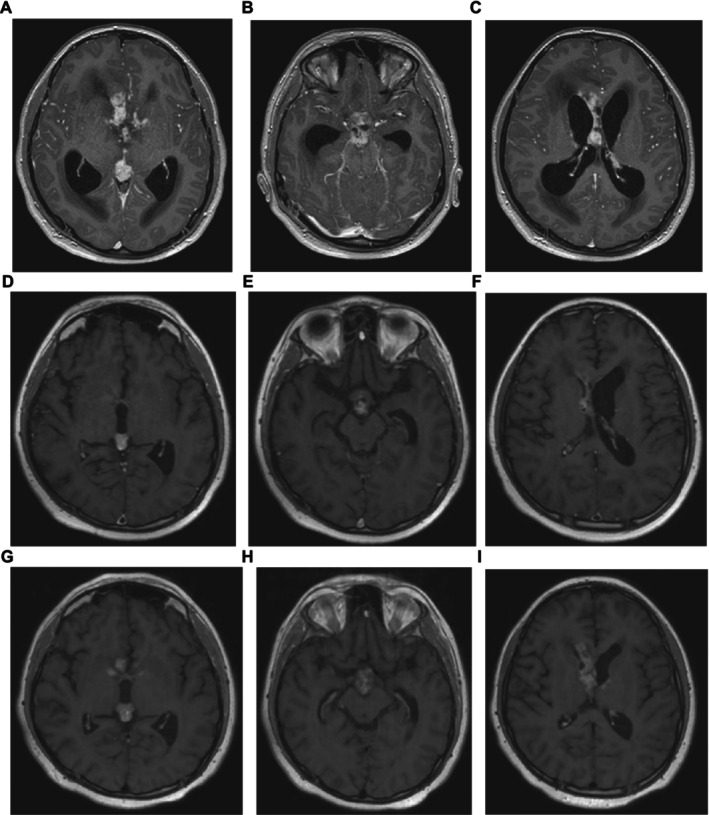
Representative radiology of the metastatic bifocal germinoma case over early time points from diagnosis. Representative axial T1‐weighted post‐contrast MRI head images. (A–C) At diagnosis, demonstrating enhancing disease in the (A) pineal, (B) suprasellar and (C) septum pellucidum regions. (D–F) At Day 9 (d9) on dexamethasone, demonstrating reduction in size and enhancement in the (D) pineal, (E) suprasellar and (F) septum pellucidum disease, presumed secondary to dexamethasone driving out the lymphocytic infiltrate from the germinoma lesions. (G–I) At Day 19 (d19) on maintenance hydrocortisone, demonstrating rebound increase in size and enhancement in the (G) pineal, (H) suprasellar and (I) septum pellucidum disease, albeit less than diagnosis, presumed secondary to lymphocytic re‐infiltration of the germinoma lesions upon cessation of dexamethasone.

Vinblastine was delivered on d0 and d7 with early MRI on d9 to assess whether the EVD could safely be removed. This demonstrated an improvement in hydrocephalus with normalisation of lateral ventricular size (Figure 1D–F). Sites of disease were reduced in both size and enhancement (Figure 1D–F), with the pineal lesion reducing to 13 × 7 × 12 mm diameter (volume 0.57 cm^3^), a tumour volume reduction of > 70% from baseline. Based on clinical (e.g., improved memory) and radiological improvement, dexamethasone was weaned and replaced by maintenance hydrocortisone (10 mg + 5 mg + 2.5 mg = 9.7 mg/m^2^/day) from d11. The EVD was retained; repeat CSF cytology from the drain on d11 was negative. Further doses of vinblastine were delivered on d13 and d19, prior to repeat MRI on d19. Unexpectedly, this demonstrated an increase in tumour size and enhancement again (Figure 1G–I), with, for example, the pineal lesion increasing to 17 × 10 × 14 mm diameter (volume 1.25 cm^3^), albeit smaller than at diagnosis. As a result, an urgent biopsy was performed on d22, along with endoscopic third ventriculostomy, Ommaya reservoir insertion, and EVD removal. Neuropathology revealed a chronic inflammatory cell infiltrate of macrophages and lymphocytes (> 95% of cells) with occasional malignant cells (< 5%) with large nuclei and prominent nucleoli (Figure 2A). Immunohistochemistry demonstrated that this dispersed cell population was positive for POU5F1 (OCT3/4) (Figure 2B) and KIT (CD117) (Figure 2C) but negative for HCG, AFP, and CD30, confirming a germinoma diagnosis.

**FIGURE 2:**
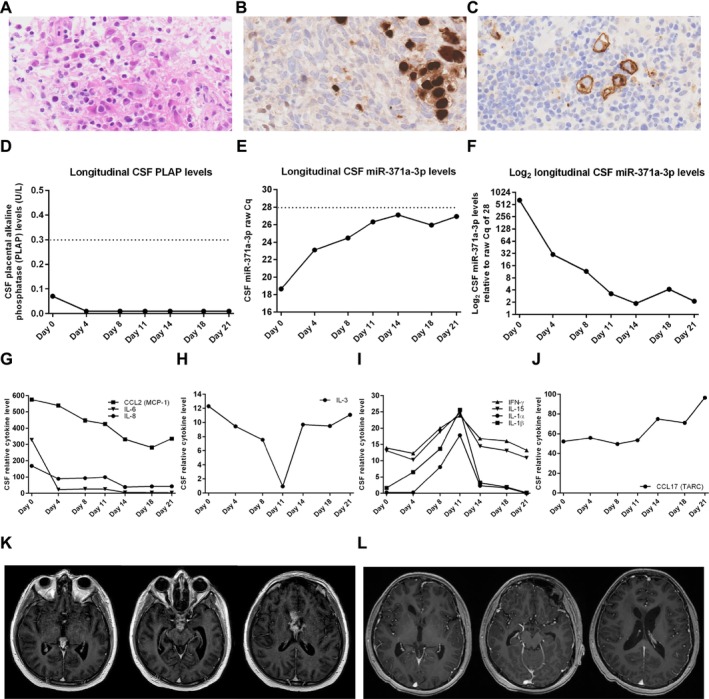
Further key features of the metastatic bifocal germinoma case, including neuropathology and circulating microRNA (miR‐371a‐3p) levels. (A–C) Neuropathology of the germinoma case, showing (A) representative haematoxylin and eosin (H&E) section showing a predominant lymphocyte infiltration (> 95% of cells) with occasional malignant cells (< 5% of total) with large nuclei and prominent nucleoli, morphologically consistent with germinoma cells. (B,C) Positive immunohistochemistry for (B) POU5F1 (OCT3/4) and C) KIT (CD117), confirming a germinoma diagnosis. (D) Longitudinal cerebrospinal fluid (CSF) placental alkaline phosphatase (PLAP) levels from d0 to d21 of vinblastine treatment, which were non‐informative/non‐diagnostic. Horizontal dotted line: diagnostic CSF PLAP threshold of 0.3 U/L. (E) Longitudinal CSF miR‐371a‐3p raw Cq PCR levels from d0 to d21 of vinblastine treatment, which were highly positive at diagnosis (raw Cq = 18.51; positive < 28) and fell with vinblastine treatment to 26.03 by d21, a > 180‐fold reduction, accurately reflecting treatment response of the germinoma disease. Horizontal dotted line: diagnostic CSF miR‐371a‐3p raw Cq threshold of 28. (F) Log_2_ longitudinal CSF miR‐371a‐3p levels relative to the raw Cq threshold of 28; any values > 1.00 are considered positive. (G–J) Longitudinal CSF levels of representative cytokines from d0 to d21 of vinblastine treatment as measured by cytokine antibody array, with relative levels (arbitrary units) obtained through normalisation (Table S1). Treatment with dexamethasone was from d‐3 to d10, which was switched to maintenance hydrocortisone on d10. (G) CCL2, IL‐6 and IL‐8 levels. (H) IL‐3 levels. (I) IL‐1α, IL‐1β, IFN‐γ and IL‐15 levels. (J) CCL17 levels. (K,L) Representative axial T1‐weighted post‐contrast MRI head images of pineal (left panel), suprasellar (middle panel) and septum pellucidum (right panel) disease: (K) pre‐protons, demonstrating partial remission, with reduction in size and enhancement of the tumours, and (L) post‐protons, demonstrating complete resolution/remission.

At the same time, the results of the circulating PLAP [8] and miR‐371a‐3p levels [11, 12, 13] became available. Diagnostic and longitudinal CSF PLAP levels were all negative (Figure 2D). Serum/CSF miR‐371a‐3p levels were quantified using a highly sensitive pre‐amplified PCR methodology our team developed [15]. More recently, we have refined the methodology such that after robust quality‐control checks, we use the raw cycle number threshold (Cq) for miR‐371a‐3p from the PCR machine to determine results, with a raw Cq threshold of < 28 denoting a positive result (i.e., meaning that miR‐371a‐3p is highly abundant) [16]. Whereas serum miR‐371a‐3p levels were negative, as may be observed [11, 12, 13], diagnostic CSF miR‐371a‐3p levels were strongly positive, with a raw Cq value of 18.51 [< 28 positive; [16]] (Figure 2E), equivalent to a diagnostic level of 717 compared with the threshold raw Cq of 28 (Figure 2F). Further, longitudinal assessment showed a response to vinblastine therapy, with d21 CSF miR‐371a‐3p down to a raw Cq of 26.03 (Figure 2E), a > 180‐fold reduction from diagnosis (Figure 2F).

In view of the early (d9 and d19) radiological changes, we hypothesised that this phenomenon was caused by the dexamethasone delivered for treatment of RICP from d‐3 to d10 resulting in the typical predominant lymphocytic infiltrate observed in germinomas being driven out and/or undergoing lympholysis due to immunosuppressive effects [5, 6, 7], which then returned following the switch to physiological maintenance hydrocortisone (d10 onwards). Accordingly, we undertook a longitudinal assessment of cytokines/chemokines in the available CSF samples using a cytokine antibody array from d0 through to d21 of vinblastine treatment (Table S1). High levels (> 30 relative units) of representative pro‐inflammatory cytokines (essential for lymphocyte/macrophage recruitment) were detected at d0, including the interleukins (IL‐) IL‐6 and IL‐8, C‐C motif chemokine ligand 2 (CCL2/MCP‐1), C‐X‐C motif chemokine ligands 1, 2, and 3 [CXCL1, CXCL2, and CXCL3—relating to growth‐regulated oncogene (GRO)‐α, GRO‐β, and GRO‐δ, respectively] and CXCL9 (MIG) (Table S1). Additionally, high levels (> 30 relative units) of representative pro‐inflammatory cytokines essential for the stimulation and recruitment of B‐lymphocytes, such as CCL8 (MCP‐2), CCL17 (TARC), CXCL12 (SDF‐1), IL‐10, and CCL22 (MDC), were also observed (Table S1). Dramatic changes in CSF cytokine levels were observed following dexamethasone (d‐3 to d10) and maintenance hydrocortisone (d10 onwards) treatment, following four main patterns. Pro‐inflammatory CCL2, IL‐6, and IL‐8 levels decreased through time and then remained at reduced levels (Figure 2G). Pro‐inflammatory IL‐3 levels decreased until d11 and then rapidly increased again (Figure 2H). IL‐1α, IL‐1β, interferon‐gamma (IFN‐γ), and IL‐15 levels increased in the CSF to d11 but then reduced again (Figure 2I). Finally, CCL17 (TARC) levels remained stable initially, but with increases after d11 (Figure 2J).

Given the histological and miR‐371a‐3p confirmation of a GCT, further vinblastine doses were delivered peripherally on d27, d33, d43, and d50 (eight doses in total). Planning MRI performed at the proton centre at d40 revealed ongoing radiological response (Figure 2K) with, for example, the pineal lesion reducing to 10 × 10 × 12 mm diameter (volume 0.63 cm^3^). Following 6 weeks of proton therapy from d60 to d95 (Phase 1: CSI 24 Gy in 15 fractions; Phase 2: tumour bed boost 16 Gy in 10 fractions), post‐proton imaging on d132 revealed complete remission, with no residual enhancing tumour demonstrated (Figure 2L).

As described here, dexamethasone treatment administered to treat RICP can result in dramatic early radiological reductions in CNS germinoma lesions [5, 6, 7]. Given these findings, accurate documentation of steroid dosing schedules used for the management of RICP in future CNS germinoma studies will be imperative to avoid overestimation of early radiological treatment responses. Histologically, both CNS germinomas and testicular seminomas (a histologically identical GCT subtype entity) are typically characterised by a profound lymphocytic infiltration, comprising ~95% of cells. Of note, the CNS, like the testis, is an immune‐privileged site. In testicular seminomas, pro‐inflammatory cytokines, such as IL‐1β, IL6, IL‐17, tumour necrosis factor‐α (TNF‐α), and IFN‐γ, are secreted by both helper (CD4+) and cytotoxic (CD8+) T‐lymphocytes, which promote an inflammatory microenvironment [17]. In addition to T‐lymphocytes, B‐lymphocytes are also abundantly found in testicular seminoma [18]. The CNS relies mainly on microglia and dendritic cells for immunosurveillance [19]. Similarly to testicular seminomas, however, CNS germinomas primarily comprise CD3+, CD4+ helper and CD8+ cytotoxic T‐lymphocytes in addition to B‐lymphocytes (including plasma cells) [20]. It has been hypothesised that the steroid‐induced phenomenon seen in this case is due to the lymphocytes in such germinoma lesions being driven out and/or undergoing lympholysis due to the immunosuppressive effects of dexamethasone [5, 6, 7], only to re‐infiltrate the tumour upon replacement with physiological maintenance hydrocortisone dosing. Here, we provide indirect evidence to support this hypothesis via our longitudinal CSF cytokine studies. At the time of diagnosis (d0), high levels of pro‐inflammatory cytokines/chemokines (e.g., IL‐6, IL‐8, IFN‐γ, CCL2, CXCL1, and CXCL9) crucial for the recruitment of lymphocytes and macrophages were detected in the CSF, as well as those that recruit B‐lymphocytes (e.g., CCL8, CCL17, CCL22, CXCL12, and IL‐10), consistent with the predominant lymphocytic infiltrate observed in germinoma. Treatment with dexamethasone for RICP resulted in longitudinal changes in cytokine levels following four main patterns (Figure 2G–J), which may well account for the steroid‐induced phenomenon described here and in other reports [5, 6, 7]. In particular, CCL2, IL‐6, and IL‐8 levels decreased longitudinally and remained low, even after dexamethasone was replaced with maintenance hydrocortisone (Figure 2G). In addition to direct immunosuppressive effects of dexamethasone on the lymphocytic infiltrate, these observed cytokine changes may also be attributable to concomitant vinblastine administration treating the germinoma cells within the lesion. Consistent with this hypothesis, just two vinblastine doses cleared the CSF of metastatic disease; further, germinoma/seminoma cells have been shown to express and secrete high levels of, for example, IL‐6 [21]. Of note, IL‐3 is produced by supporting astrocytes in the CNS as well as by activated CD4+ T‐lymphocytes in inflammatory conditions [22]. The reduction of IL‐3 levels with dexamethasone before rapidly increasing again upon its cessation (Figure 2H) is consistent with immunosuppressive effects of dexamethasone on this lymphocyte subtype. Interestingly, the pro‐inflammatory cytokines IL‐1α, Il‐1β, IFN‐γ, and IL‐15 increased in abundance during dexamethasone treatment but then reduced again (Figure 2I). These cytokines play a pivotal role in immune cell recruitment in seminomas [18], and it is therefore feasible that the initial increases observed in these modestly abundant cytokines were a rebound feedback mechanism to the dramatic reductions in the highly abundant CCL2, IL‐6, and IL‐8 cytokines caused by dexamethasone, which then fell again upon increases in other cytokines such as IL‐3 (Figure 2H) and CCL17 (Figure 2J), which recruits and maintains B‐lymphocytes [23]. These data suggest that the balance of various immune cell subsets in the lymphocytic population present within the germinoma lesion may have evolved over time, potentially moving towards a B‐lymphocyte maintenance response rather than a cytotoxic T‐lymphocyte response.

Although a low threshold for undertaking biopsy of bifocal cases should be maintained if any presenting features are atypical, the clinical and radiological features here, along with negative AFP/HCG markers in both compartments, confirmed the European recommendation that treatment could be initiated without histological confirmation [3], supported by review of a large bifocal series [4]. The radiological rebound that occurred on switching dexamethasone for RICP back to maintenance hydrocortisone did result in careful multidisciplinary team discussion and the decision to biopsy, which indeed confirmed germinoma, allowing the planned treatment to continue, with bridging vinblastine monotherapy [11, 14], prior to CSI. The vinblastine approach also allowed an EVD/septostomy to be performed, rather than placement of a ventriculoperitoneal (VP) shunt, which have been associated with intra‐abdominal metastasis via shunt seeding in other metastatic germinoma cases treated with CSI alone, without systemic chemotherapy [24]. Of note, the positive CSF at diagnosis had cleared by re‐assessment at d11 after just two vinblastine doses. Further, use of vinblastine in metastatic germinoma cases prior to definitive radiotherapy may help to prevent the development of further co‐morbidities [11, 14] and is being explored in the upcoming UK ‘MonoGerm’ trial [25].

Although PLAP quantification has been proposed as being effective at diagnosing intracranial GCTs, and germinoma in particular [8], we have not found this to be helpful. Indeed, in this bulky metastatic disease case, as in others we have profiled, levels were completely non‐diagnostic. In contrast, circulating miR‐371a‐3p quantification has shown substantial promise [11, 12, 13], also confirmed here. CSF miR‐371a‐3p levels were strongly positive at diagnosis and fell rapidly during the first 3 weeks of vinblastine treatment to d21, whereas the disease reduced and then increased again radiologically due to presumed changes in lymphocytic infiltration. These data show that miR‐371a‐3p levels accurately reflect true germinoma/GCT disease activity, as has been shown for extracranial counterparts [12, 26, 27, 28]. Of note, a high detection rate of CSF circulating tumour DNA in CNS GCTs has recently been reported, and this may also offer additional diagnostic certainty in such cases [29].

In summary, we present an interesting patient presenting with metastatic bifocal germinoma in whom there were some key learning points: firstly, the dramatic early radiological response to steroids and rebound following discontinuation, likely underpinned mechanistically by alterations in immunosuppressive effects, assessed by cytokine changes within the CSF over time; secondly, the utility of circulating miR‐371a‐3p quantification for malignant GCT diagnosis and monitoring; and thirdly, the use of vinblastine monotherapy prior to definitive CSI to avoid the development of further co‐morbidities. Reporting such learning points will facilitate the delivery of optimal care for such patients and continue to allow improved clinical outcomes.

## Author Contributions

Study concept: C.G.S. and M.J.M. Data curation, analysis and interpretation: C.G.S., D.W. and M.J.M. Clinical input: P.P., I.J., N.T., B.G.F., A.E.J.H., L.S., J.C.N., T.A., G.H., G.A.A.B., N.C., K.A. and M.J.M. Manuscript writing: C.G.S. and M.J.M. Manuscript revision and approval: C.G.S., D.W., P.P., I.J., N.T., B.G.F., A.E.J.H., L.S., J.C.N., T.A., G.H., G.A.A.B., N.C., K.A. and M.J.M.

## Conflicts of Interest

The authors declare no conflicts of interest.

## Supporting information


**Table S1.** Longitudinal cytokine levels over time [Day 0 (d0) through to d21 of vinblastine treatment] in the cerebrospinal fluid (CSF) of the patient following dexamethasone treatment (d‐3 to d10) and then physiological maintenance hydrocortisone treatment (d11 onwards). The Human Cytokine Antibody Array (catalogue number ab133997, Abcam, Cambridge, UK) was used to quantify the levels of 42 cytokines/chemokines. For this work, 500 μL of CSF for each of the time points was used and diluted 1:1 with an equal volume of blocking buffer provided in the kit, and the array run as per the manufacturer’s recommendations on the provided array membranes. Following incubation, for each time‐point, the chemiluminescent signal corresponding to each of the 42 cytokines on the membrane was captured using Azure BioSystems C600 Western Blot Imaging System (Azure BioSystems, Dublin, CA, USA), and the obtained images were then quantified by using IMAGEJ software (https://imagej.net) to determine the densitometry of each individual cytokine. Next, normalisation was performed by subtracting the densitometry values of the negative controls provided on each membrane. The resultant normalised densitometry values were then referenced to the positive controls on each membrane, as per the manufacturer’s recommendations, resulting in levels reported in relative arbitrary units. This approach allowed direct comparison between levels of different cytokines at all assessed time‐points. Cytokines in the table were ranked by levels on d0. High levels on d0 were arbitrarily defined as those cytokines with an expression value of > 30 relative units (i.e., 12 of the 42 interrogated cytokines). Note the presence of some negative expression values for a small number of low‐ranking (i.e., low abundant) cytokines at some time‐points, due to the normalisation to negative controls, as described above.

## Data Availability

The raw data used to generate the results in this manuscript are available upon reasonable request.
