# Supplementary material for: Metastatic Bifocal Germinoma With Dramatic Early Steroid Response, Utility of Circulating miR‐371a‐3p and Vinblastine Monotherapy Prior to Definitive Craniospinal Irradiation
Source: Neuropathol Appl Neurobiol. 2025 Jul 5;51(4):e70030. doi: 10.1111/nan.70030 (PMC12228120; doi:10.1111/nan.70030)
Supplement: Supplementary file 1 — Table S1. Longitudinal cytokine levels over time [Day 0 (d0) through to d21 of vinblastine treatment] in the cerebrospinal fluid (CSF) of the patient following dexamethasone treatment (d‐3 to d10) and then physiological maintenance hydrocortisone treatment (d11 onwards). The Human Cytokine Antibody Array (catalogue number ab133997, Abcam, Cambridge, UK) was used to quantify the levels of 42 cytokines/chemokines. For this work, 500 μL of CSF for each of the time points was used and diluted 1:1 with an equal volume of blocking buffer provided in the kit, and the array run as per the manufacturer’s recommendations on the provided array membranes. Following incubation, for each time‐point, the chemiluminescent signal corresponding to each of the 42 cytokines on the membrane was captured using Azure BioSystems C600 Western Blot Imaging System (Azure BioSystems, Dublin, CA, USA), and the obtained images were then quantified by using IMAGEJ software (https://imagej.net) to determine the densitometry of each individual cytokine. Next, normalisation was performed by subtracting the densitometry values of the negative controls provided on each membrane. The resultant normalised densitometry values were then referenced to the positive controls on each membrane, as per the manufacturer’s recommendations, resulting in levels reported in relative arbitrary units. This approach allowed direct comparison between levels of different cytokines at all assessed time‐points. Cytokines in the table were ranked by levels on d0. High levels on d0 were arbitrarily defined as those cytokines with an expression value of > 30 relative units (i.e., 12 of the 42 interrogated cytokines). Note the presence of some negative expression values for a small number of low‐ranking (i.e., low abundant) cytokines at some time‐points, due to the normalisation to negative controls, as described above. [file NAN-51-e70030-s001.docx]

**Supplementary Table S1**

| **Rank** | **Cytokine/Chemokine** | **Day 0 (d0)** | **Day 4 (d4)** | **Day 8 (d8)** | **Day 11 (d11)** | **Day 14 (d14)** | **Day 18 (d18)** | **Day 21 (d21)** |
| --- | --- | --- | --- | --- | --- | --- | --- | --- |
| 1 | CCL2 (MCP-1) | 575.2982 | 538.2491 | 447.2554 | 425.7476 | 331.2858 | 280.7491 | 335.4933 |
| 2 | ANGIOGENIN | 397.4891 | 169.4507 | 211.8421 | 209.0514 | 147.6668 | 144.204 | 219.3108 |
| 3 | IL-6 | 327.5178 | 22.57461 | 26.76326 | 25.8083 | 5.3072 | 4.798275 | 3.715494 |
| 4 | IL-8 | 168.9771 | 89.23447 | 93.46662 | 99.45095 | 38.24406 | 42.44901 | 42.61721 |
| 5 | CXCL9 (MIG) | 162.2734 | 26.23654 | 42.50949 | 68.25303 | 36.93792 | 44.00065 | 62.06656 |
| 6 | CXCL1/2/3 (GRO) | 61.82037 | 10.8749 | 17.12498 | 17.84039 | 6.319879 | 3.436432 | 8.855191 |
| 7 | CCL8 (MCP-2) | 53.94925 | 33.51195 | 23.66358 | 29.63371 | 32.19896 | 28.78977 | 35.07738 |
| 8 | CCL17 (TARC) | 52.16025 | 55.87069 | 49.52519 | 53.41233 | 74.93988 | 71.01514 | 96.51569 |
| 9 | CXCL12 (SDF-1) | 37.33789 | 42.75286 | 50.54734 | 40.82133 | 36.92408 | 37.79831 | 61.54192 |
| 10 | CCL22 (MDC) | 36.59575 | 15.70789 | 19.95266 | 21.188 | 13.29482 | 15.52663 | 22.48019 |
| 11 | IGF-I | 33.12311 | 17.57244 | 17.18099 | 12.31863 | 17.84293 | 15.72931 | 23.52223 |
| 12 | IL-10 | 30.75839 | 13.44218 | 17.73046 | 15.22253 | 7.745271 | 6.812265 | 8.03031 |
| 13 | IL-7 | 29.91685 | 8.890476 | 14.36085 | 11.94663 | 3.739237 | 6.703857 | 6.385937 |
| 14 | OSM | 25.1505 | 14.8951 | 22.71651 | 32.70788 | 15.63123 | 21.10559 | 24.68999 |
| 15 | CCL7 (MCP-3) | 21.76493 | 11.10193 | 6.909234 | 8.948142 | 8.65101 | 7.186669 | 7.795304 |
| 16 | IL-5 | 19.79557 | 4.924716 | 6.043222 | 6.093901 | 2.597214 | 2.962598 | 0.41401 |
| 17 | CCL5 (RANTES) | 17.87301 | 12.78407 | 18.66971 | 11.12681 | 8.507358 | 13.06238 | 15.42347 |
| 18 | IL-2 | 17.57772 | 9.250628 | 6.93057 | 9.444433 | 8.438862 | 6.882475 | 6.77692 |
| 19 | CCL15 (MIP-1δ) | 17.06405 | 8.325017 | 12.55922 | 12.41758 | 6.411122 | 11.18026 | 12.90247 |
| 20 | ENA-79 | 17.05261 | 5.317714 | 9.510822 | 9.493511 | 1.897963 | 3.461001 | 4.259592 |
| 21 | TGF-β1 | 16.99187 | 17.09636 | 23.83221 | 20.4327 | 15.20827 | 16.08846 | 15.39717 |
| 22 | MCSF | 16.0144 | 13.0672 | 12.51266 | 15.18712 | 7.798717 | 9.287653 | 9.979032 |
| 23 | KITLG (SCF) | 14.86387 | 17.81857 | 23.7105 | 18.33026 | 11.48148 | 13.52171 | 16.97053 |
| 24 | IFN-γ | 13.89919 | 12.26652 | 19.97993 | 23.85233 | 16.8031 | 16.01265 | 13.20917 |
| 25 | IL-15 | 13.08367 | 10.2965 | 18.88778 | 24.62487 | 14.41127 | 13.08399 | 10.88869 |
| 26 | IL-3 | 12.29662 | 9.453743 | 7.557842 | 0.940113 | 9.70002 | 9.499311 | 11.07214 |
| 27 | LEPTIN | 11.15336 | 9.803476 | 20.91271 | 19.25038 | 12.07564 | 11.84742 | 14.00751 |
| 28 | VEGF | 10.7291 | 7.912803 | 15.12829 | 9.340391 | 8.159088 | 10.01994 | 10.24717 |
| 29 | PDGF BB | 9.835032 | 9.53096 | 17.13517 | 14.12216 | 5.473373 | 6.6618 | 7.426176 |
| 30 | TNF-α | 8.593412 | 12.54883 | 3.153564 | 0.778978 | 19.37377 | 9.938453 | 4.56315 |
| 31 | IL-4 | 8.075024 | 4.341556 | 3.674173 | 3.480172 | 5.659632 | 6.359685 | 4.420844 |
| 32 | THROMBOPOIETIN | 7.177362 | 3.98193 | 11.95265 | 8.655684 | 3.780515 | 7.744146 | 6.961464 |
| 33 | IL-12 | 5.925609 | 9.007439 | 13.28195 | 13.28349 | 6.851155 | 5.743592 | 4.790978 |
| 34 | CXCL1 (GRO-α) | 5.284348 | 0.664252 | 8.368319 | 12.26965 | -0.91656 | -2.23201 | -4.30093 |
| 35 | TNF-β | 3.149585 | 5.513954 | 2.78033 | 0.938392 | 10.60018 | 5.390612 | 4.606371 |
| 36 | IL-13 | 3.097212 | 3.994183 | 9.276392 | 12.11235 | 5.23196 | 3.874448 | 2.185929 |
| 37 | EGF | 1.836054 | 6.082391 | 4.537274 | 0.581737 | 5.850894 | 3.12939 | 6.08678 |
| 38 | IL-1β | 1.653692 | 6.518307 | 13.63949 | 25.57682 | 3.21521 | 1.911272 | -1.48499 |
| 39 | GM-CSF | 0.520067 | -1.48987 | 3.717017 | 0.124836 | -1.76737 | -1.86146 | -1.21166 |
| 40 | IL-1α | 0.289576 | 0.256269 | 8.052054 | 17.82829 | 2.311361 | 1.680132 | -0.71702 |
| 41 | CCL1 (I-309) | -0.81051 | -0.55041 | 6.436923 | 11.39233 | 0.577168 | -0.45441 | -3.43148 |
| 42 | GCSF | -1.39485 | -1.78 | 3.000158 | -0.71245 | -2.2284 | -2.4451 | -2.2633 |

**Supplementary Table S1**. **Longitudinal cytokine levels over time [Day 0 (d0) through to d21 of vinblastine treatment] in the cerebrospinal fluid (CSF) of the patient following dexamethasone treatment (d-3 to d10) and then physiological maintenance hydrocortisone treatment (d11 onwards).** The Human Cytokine Antibody Array (catalogue number ab133997, Abcam, Cambridge, UK) was used to quantify the levels of 42 cytokines/chemokines. For this work, 500µl of CSF for each of the time-points was used and diluted 1:1 with an equal volume of blocking buffer provided in the kit, and the array run as per the manufacturer’s recommendations on the provided array membranes. Following incubation, for each time-point, the chemiluminescent signal corresponding to each of the 42 cytokines on the membrane was captured using Azure BioSystems C600 Western Blot Imaging System (Azure BioSystems, Dublin, CA, USA), and the obtained images were then quantified by using IMAGEJ software (<https://imagej.net>) to determine the densitometry of each individual cytokine. Next, normalisation was performed by subtracting the densitometry values of the negative controls provided on each membrane. The resultant normalised densitometry values were then referenced to the positive controls on each membrane, as per the manufacturer’s recommendations, resulting in levels reported in relative arbitrary units. This approach allowed direct comparison between levels of different cytokines at all assessed time-points. Cytokines in the Table were ranked by levels on d0. High levels on d0 were arbitrarily defined as those cytokines with an expression value of >30 relative units (i.e., 12 of the 42 interrogated cytokines). Note the presence of some negative expression values for a small number of low-ranking (i.e., low abundant) cytokines at some time-points, due to the normalisation to negative controls, as described above.
